# Editorial: Nutritional Intervention in Cardiovascular Diseases: From Basic Science to Applied Investigations

**DOI:** 10.3389/fphys.2022.858346

**Published:** 2022-03-04

**Authors:** Vladimir Lj. Jakovljevic, Dragan M. Djuric, Naranjan S. Dhalla

**Affiliations:** ^1^Department of Physiology, Faculty of Medical Sceinces, University of Kragujevac, Kragujevac, Serbia; ^2^Department of Human Pathology, I.M. Sechenov First Moscow State Medical University, Moscow, Russia; ^3^School of Medicine, Institute of Medical Physiology “Richard Burian”, University of Belgrade, Belgrade, Serbia; ^4^St. Boniface Hospital Albrechtsen Research Centre, Institute of Cardiovascular Sciences, University of Manitoba, Winnipeg, MB, Canada

**Keywords:** nutrition, cardiovascular diseases, metabolism, basic investigations, clinical investigation

A modern lifestyle, characterized by increasing requirements that an individual must meet in order to fit into everyday routine, results in a reduction of time that a person can devote to himself and his health. Furthermore, a modern man confronts with evolutionary mismatch due to sedentary lifestyle and unlimited intake of high-calorie foods. During the long history of human species development, physical ability and frugality of the body in the consumption of caloric reserves represented a kind of evolutionary advantage. Today, these evolutionary advantages combined with the lack of free time, favoritism of high-calorie meals, increased stress and reduced physical activity, present one of the main causes of the burden of non-communicable diseases, mainly affecting cardiovascular system.

Cardiovascular diseases (CVD) are the leading cause of death worldwide in both developed and developing countries. Although various pharmacological strategies are available and are constantly evolving in the treatment of CVD, the global burden of CVD remains constantly high. Given that CVD are preventable and that various risk factors (such as smoking, sedentary lifestyle and unhealthy diet) have been identified, increasing attention is being paid to different modalities of prevention (Kaminsky et al., 2021; Soltani et al., 2021). Improving nutritional status, either by reducing caloric intake or by improving the quality of food (in terms of vitamins, minerals, reduced salt and trans-fatty acids) is an effective way to prevent CVD (Shah and Dhalla, 2021; Stadler and Marsche, 2021). Due to complex etiology of CVD and various pathophysiological mechanisms involved, different nutritional interventions may target some specific pathological factors (oxidative stress or inflammation) or etiological factors (Liu et al., 2018; Mattavelli et al., 2021; Hariharan et al., 2022). In this context, the aim of this Research Topic was to bring novel information regarding nutritional interventions in the prevention and treatment of CVD.

Gut dysbiosis appears to be involved in pathophysiology of various types of CVD through different mechanisms and molecules (Yang et al., 2021). On this topic, Singh et al. have shown that high-fat diet (HFD) enteric microbiome composition and induced changes in activity of several enzymes involved in the regulation of molecules important in maintaining cardiovascular homeostasis. In particular, the remodeling of cardiac tissue and multiple organ damage were accompanied by altered activity of enzymes involved in metabolism of 1-carbon and homocysteine by HFD. All these adverse effects of HFD and gut dysbiosis were significantly alleviated by probiotic (*Lactobacillus rhamnosus*). Probiotic treatment also induced browning of white adipose tissue and improvement of heart function. In another article, Stanisic et al. have confirmed that gut microbiota alterations provoked by HFD showed significant increase in body weight as well as increased blood glucose values. The levels of pro-inflammatory cytokines and mediators (TNF-α, IL-1β, IL-6, TLR4, MMP-2, and MMP-9) were significantly increased in periodontal tissue and reduced gingival blood flow by HFD and dysbiosis. Probiotic *Lactobacillus rhamnosus* treatment resulted in significant reduction of pro-inflammatory environment and associated changes in oral tissues and improved gingival blood flow.

Phytomedicine is an increasingly common method of disease prevention and supportive therapy. Active principles derived from various plants show different potential in treatment of CVD. Draginic et al. have reviewed the current literature about possibilities and expediency of using lemon balm (*Melissa officinalis*) in the treatment of CVD. The presence of flavonoids and other compounds with strong antioxidative properties in lemon balm probably forms the basis of its therapeutic effect. The beneficial effects including sedative, hypoglycemic, hepatoprotective, antibacterial, anti-inflammatory, antioxidant, antiviral, antispasmodic, and neuroprotective actions are noteworthy. Mild antiarrhythmic effects of lemon balm were confirmed in different *in vitro, in vivo*, and *ex vivo* experimental models, mainly mediated by slowing the conduction of impulses through the heart. The authors also highlighted the vasorelaxant effects and anti-inflammatory action of lemon balm, as well as cardioprotective activity in various cardiometabolic states.

Due to crucial role of vitamins in regulation of various metabolic pathways and maintaining overall cardiovascular homeostasis, Shah and Dhalla have reviewed the role of vitamin deficiency for occurrence of CVD, as well as possibility of vitamin supplementation in prevention and treatment. Regarding hydro-soluble vitamins, deficit of vitamin B_1_, B_6_, B_12_, and C seems to be associated with occurrence of cardiovascular disorders, while, of the lipo-soluble vitamins, vitamin D is most important for maintaining cardiovascular health. Pretreatment with vitamins attenuated damage of the heart in an experimental model of ischemia/reperfusion (I/R) injury or mitigated catecholamine-induced ventricular arrhythmias. On the other hand, clinical trials showed that vitamin therapy had low or no impact on developed cardiovascular disorders. It was concluded that vitamin supplementation could be more effective in the prevention than the treatment of CVD, except in patients with hypovitaminosis.

Savencu et al. reviewed the results of investigations dealing with different approaches in the prevention and treatment of CVD. Caloric restriction (CR) and intermittent fasting (IF) are two strategies of caloric restriction able to improve cardiovascular health and cardiovascular metabolism. CR and IF improved mitochondrial ultrastructure and dynamics, reduced mitochondria-related production of reactive oxygen species (ROS), as well as improved antioxidative ability and mitochondrial respiration. Various regimes of CR improved different aspects of heart metabolic function and increased insulin sensitivity, reduced fibrosis, apoptosis and hypertrophy, and promoted endothelium-dependent vasorelaxation. Investigations dealing with effects of various modalities of IF on the heart also showed protective role in many pathological conditions such as myocardial infarction, endothelial dysfunction or insulin resistance.

Since the aim of these topics in this Research Topic was to present experimental and clinical information regarding the effects of known and novel dietary supplements and dietary regimes for the reduction of risk of CVD, it is hoped that these articles in this Research Topic meet the expectations of our readers which may greatly help researchers who are planning novel investigations in this area. Nonetheless, we declare that the preparations of this Research Topic were conducted in the absence of any commercial or financial relationships that could be construed as a potential conflict of interest. We are also grateful to all authors for submitting their inspiring papers to this Research Topic and all the reviewers for having provided their valuable contribution to improve the quality of each manuscript.

## Author Contributions

All authors listed have made a substantial, direct, and intellectual contribution to the work and approved it for publication.

## Conflict of Interest

The authors declare that the research was conducted in the absence of any commercial or financial relationships that could be construed as a potential conflict of interest.

## Publisher's Note

All claims expressed in this article are solely those of the authors and do not necessarily represent those of their affiliated organizations, or those of the publisher, the editors and the reviewers. Any product that may be evaluated in this article, or claim that may be made by its manufacturer, is not guaranteed or endorsed by the publisher.
